# Supplementary material for: Eosinophil-epithelial interactions mediate protective intestinal remodeling during food allergy
Source: bioRxiv. 2026 Jun 8:2026.06.04.730224. Preprint. [Version 1] doi: 10.64898/2026.06.04.730224 (PMC13278080; doi:10.64898/2026.06.04.730224)
Supplement: Supplementary file 1 [file NIHPP2026.06.04.730224v1-supplement-1.pdf]

bioRxiv preprint doi: https://doi.org/10.64898/2026.06.04.730224; this version posted June 8, 2026. The copyright holder for this preprint (which was not certified by peer review) is the author/funder, who has granted bioRxiv a license to display the preprint in perpetuity. It is made available under a CC-BY 4.0 International license.

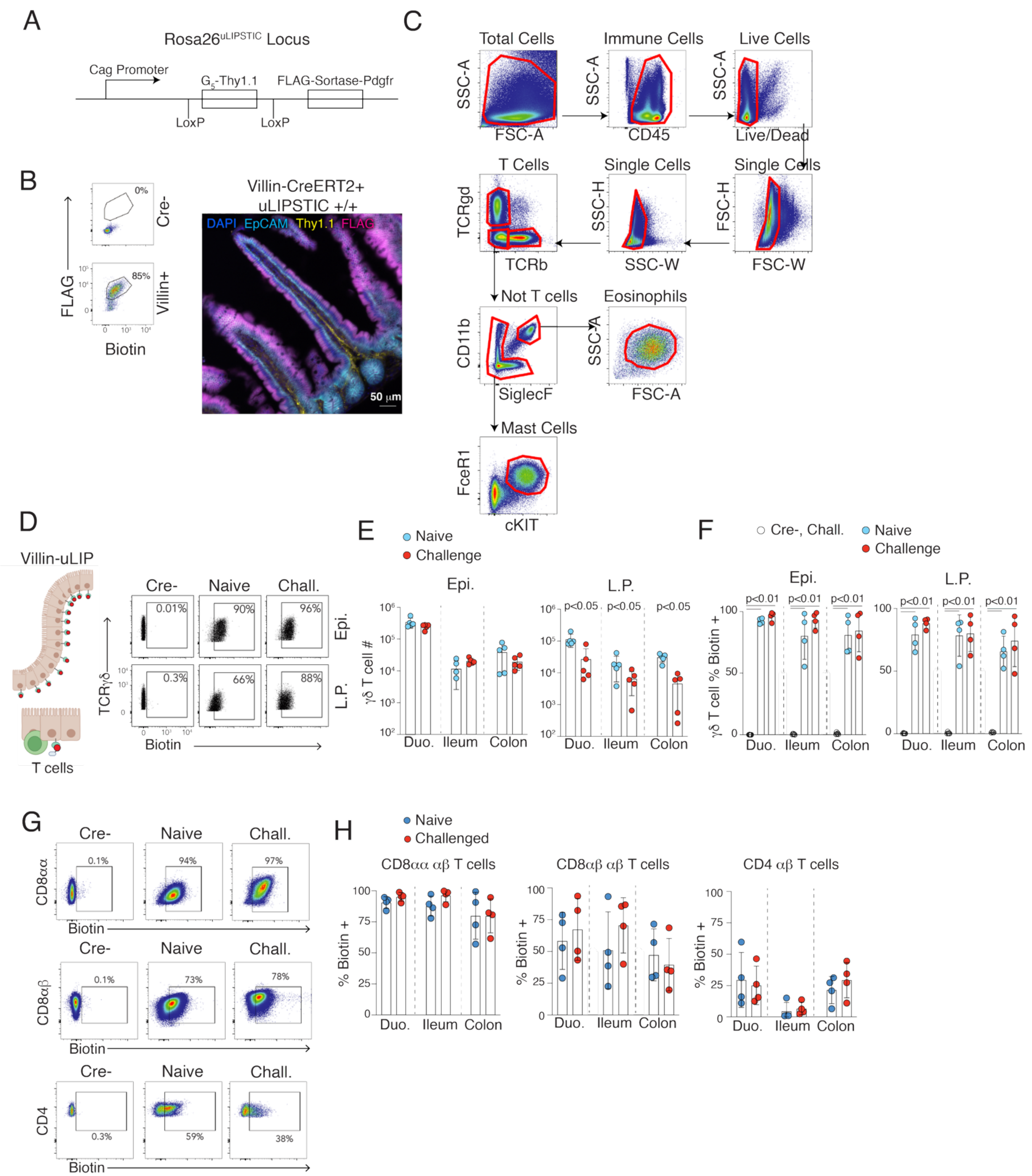

Supplemental Figure 1. uLIPSTIC technology validation and gating strategies. A. Schematic representation of the Rosa26-uLIPSTIC allele, showing the genetic construct used for sortase-mediated

bioRxiv preprint doi: https://doi.org/10.64898/2026.06.04.730224; this version posted June 8, 2026. The copyright holder for this preprint (which was not certified by peer review) is the author/funder, who has granted bioRxiv a license to display the preprint in perpetuity. It is made available under a CC-BY 4.0 International license.

labeling. **B**. Validation of Villin-uLIPSTIC functionality. Left: Flow cytometry staining of epithelial cells isolated from Cre- (negative control) and Villin-CreERT2+ uLIPSTIC +/+ mice for FLAG (tag bound to sortase) and Biotin (LPETG-Biotin substrate). Bottom: Representative confocal image of Villin-CreERT2+ uLIPSTIC +/+ epithelium (DAPI-dark blue, EpCAM-light blue, FLAG-magenta, Thy1.1-yellow). **C**. Flow cytometry gating schemes used throughout the study to identify γδ T cells (CD45+, TCRγδ+, TCRβ−), αβ T cells (CD45+, TCRγδ−, TCRβ+), mast cells (CD45+, TCRγδ−, TCRβ−, cKIT+, Fcer1+), and eosinophils (CD45+, TCRγδ−, TCRβ−, CD11b+, SiglecF+, SSC-Ahi). **D-H**. Flow cytometry analysis comparing biotin labeling between control and uLIPSTIC mice at steady state. **D-G**, Representative flow cytometry plots of biotin labeling in Cre- uLIPSTIC +/+ mice after the sixth OVA-OC (left), Villin-CreERT2+ uLIPSTIC +/+ challenge-naïve mice (middle), and Villin-CreERT2+ uLIPSTIC +/+ mice after the sixth OVA-OC (right), showing γδ T cells (**D**), CD8αα T cells (**E** top), CD8αβ T cells (**E** middle), and CD4 T cells (**E** bottom, shown in panel **G**). **H**, Summary data from n=4 mice per group (**E-H**). Welch's T test was performed; no significant differences were observed between control and uLIPSTIC mice at baseline. Each dot in the bar graphs represents a biological replicate derived from a unique mouse, and the error bars represent the standard deviation.

bioRxiv preprint doi: https://doi.org/10.64898/2026.06.04.730224; this version posted June 8, 2026. The copyright holder for this preprint (which was not certified by peer review) is the author/funder, who has granted bioRxiv a license to display the preprint in perpetuity. It is made available under a CC-BY 4.0 International license.

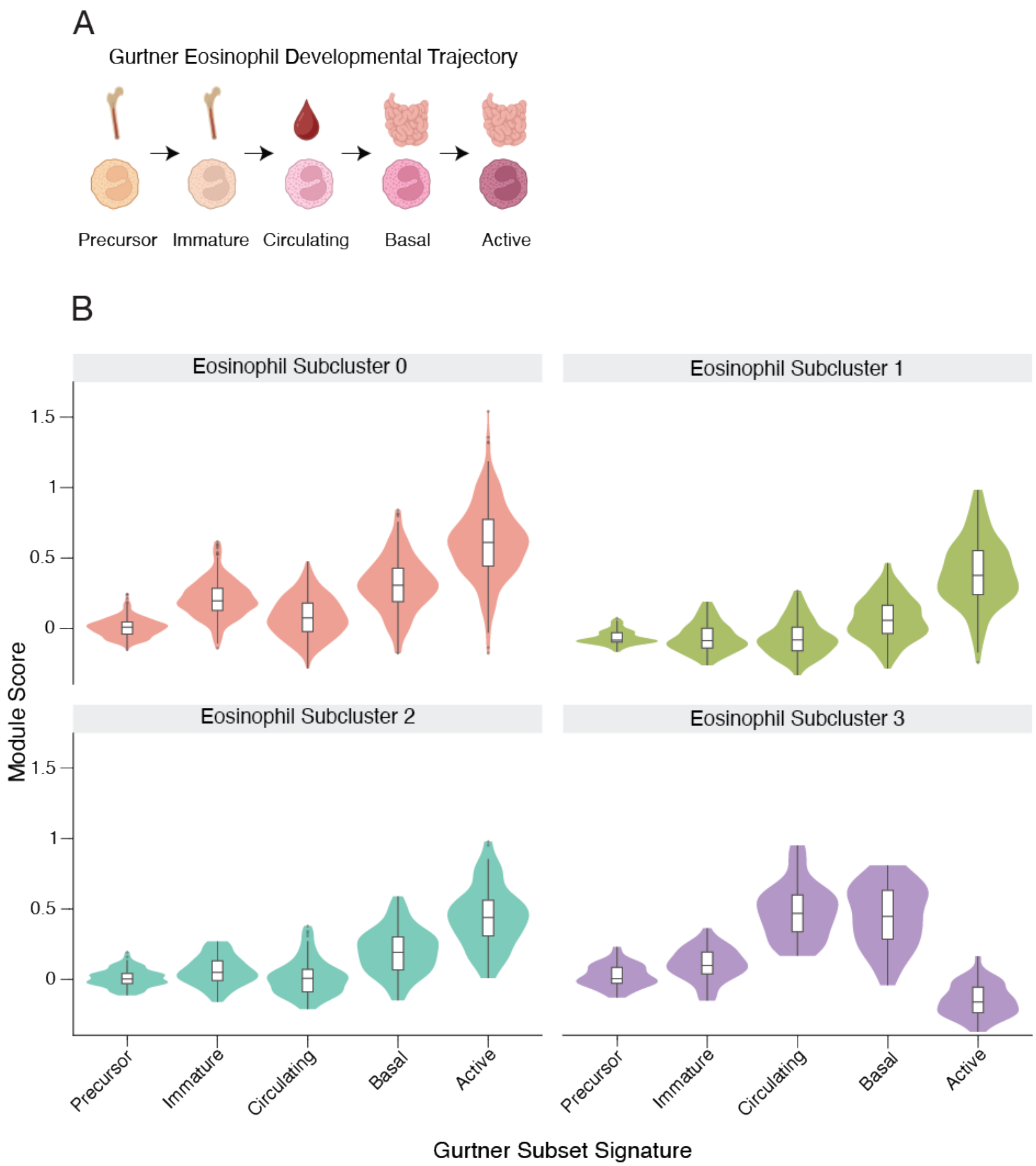

bioRxiv preprint doi: https://doi.org/10.64898/2026.06.04.730224; this version posted June 8, 2026. The copyright holder for this preprint (which was not certified by peer review) is the author/funder, who has granted bioRxiv a license to display the preprint in perpetuity. It is made available under a CC-BY 4.0 International license.

Supplemental Figure 2. Eosinophil developmental state analysis. A. Schematic depicting the eosinophil developmental and differentiation trajectory proposed by Gurtner et al., illustrating the progression from immature to mature and active eosinophil states. **B**. Module score analysis of eosinophil developmental states. For each eosinophil cluster defined in Figure 3C, a gene signature corresponding to the EO_active population (derived from Gurtner et al.) was used to compute the module scores in the single-cell RNA-seq dataset. The gene lists were filtered to include only those genes that were detected in the dataset. Per-cell module scores were calculated using Seurat's AddModuleScore function on log-normalized RNA expression data, which computes the average expression of the gene set subtracted from the expression of matched control gene sets to account for technical variation. Cells were grouped into Seurat-defined clusters, and module score distributions were visualized using violin plots. The module scores were summarized per cluster by calculating the mean, standard deviation, and cell count. Statistical differences in module scores across clusters were assessed using Kruskal-Wallis test, followed by pairwise Wilcoxon rank-sum tests with Benjamini-Hochberg correction for multiple comparisons.

bioRxiv preprint doi: https://doi.org/10.64898/2026.06.04.730224; this version posted June 8, 2026. The copyright holder for this preprint (which was not certified by peer review) is the author/funder, who has granted bioRxiv a license to display the preprint in perpetuity. It is made available under a CC-BY 4.0 International license.

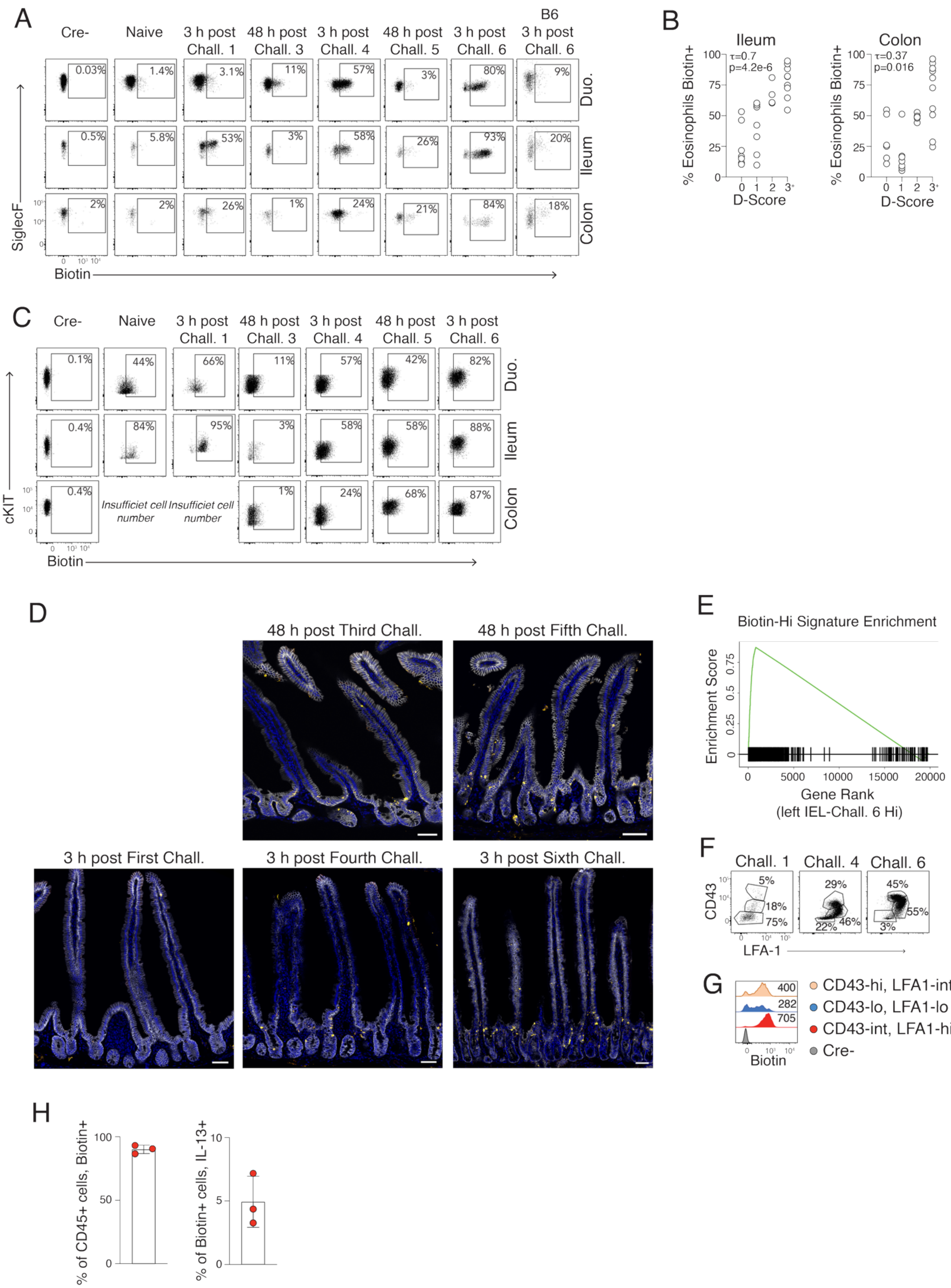

bioRxiv preprint doi: https://doi.org/10.64898/2026.06.04.730224; this version posted June 8, 2026. The copyright holder for this preprint (which was not certified by peer review) is the author/funder, who has granted bioRxiv a license to display the preprint in perpetuity. It is made available under a CC-BY 4.0 International license.

Supplemental Figure 3. Kinetic and regional analyses of immune-epithelial interactions. A. Representative flow cytometry plots of Villin-uLIPSTIC biotin labeling of eosinophils (CD45+, TCRγδ-, TCRβ-, CD11b+, SiglecF+) at the indicated challenge time points. **B**. Correlation analysis of diarrhea score with Villin-uLIPSTIC biotin labeling of eosinophils in the ileum (left, $\tau=0.7$, $p=4.2e-6$) and colon (right, $\tau=0.37$, $p=0.016$). The Jonckheere-Terpstra test was performed. Each dot represents a biological replicate obtained from a unique mouse. **C**. Representative flow cytometry plots of Villin-uLIPSTIC biotin labeling of mast cells (CD45+, TCRγδ-, TCRβ-, c-KIT +, Fcer1+) at the indicated challenge time points. **D**. Confocal imaging of mast cells in the duodenum at the indicated challenge timepoints (DAPI-dark blue, EpCAM-white, Mcpt1-yellow). **E**. Gene set enrichment analysis comparing the Biotin-Hi eosinophil gene expression signature defined in the single-cell RNA-seq dataset to the bulk eosinophil RNA-seq dataset from Figure 4H. **F-G**. Functional characterization of eosinophil subsets. **F**. Representative flow cytometry plots depicting CD43 and LFA-1 staining of eosinophils at the indicated challenge time points. **G**. Villin-uLIPSTIC biotin labeling of indicated eosinophil subsets at sixth OVA-OC, stratified by CD43/LFA-1 expression. **H**. Quantification of Villin-uLIPSTIC biotin labeling. Left: Absolute number of biotin + immune cells. Right: Number of biotin+ IL-13+ cells. Each dot represents a biological replicate derived from a unique mouse, and the error bars represent the standard deviation.

bioRxiv preprint doi: https://doi.org/10.64898/2026.06.04.730224; this version posted June 8, 2026. The copyright holder for this preprint (which was not certified by peer review) is the author/funder, who has granted bioRxiv a license to display the preprint in perpetuity. It is made available under a CC-BY 4.0 International license.

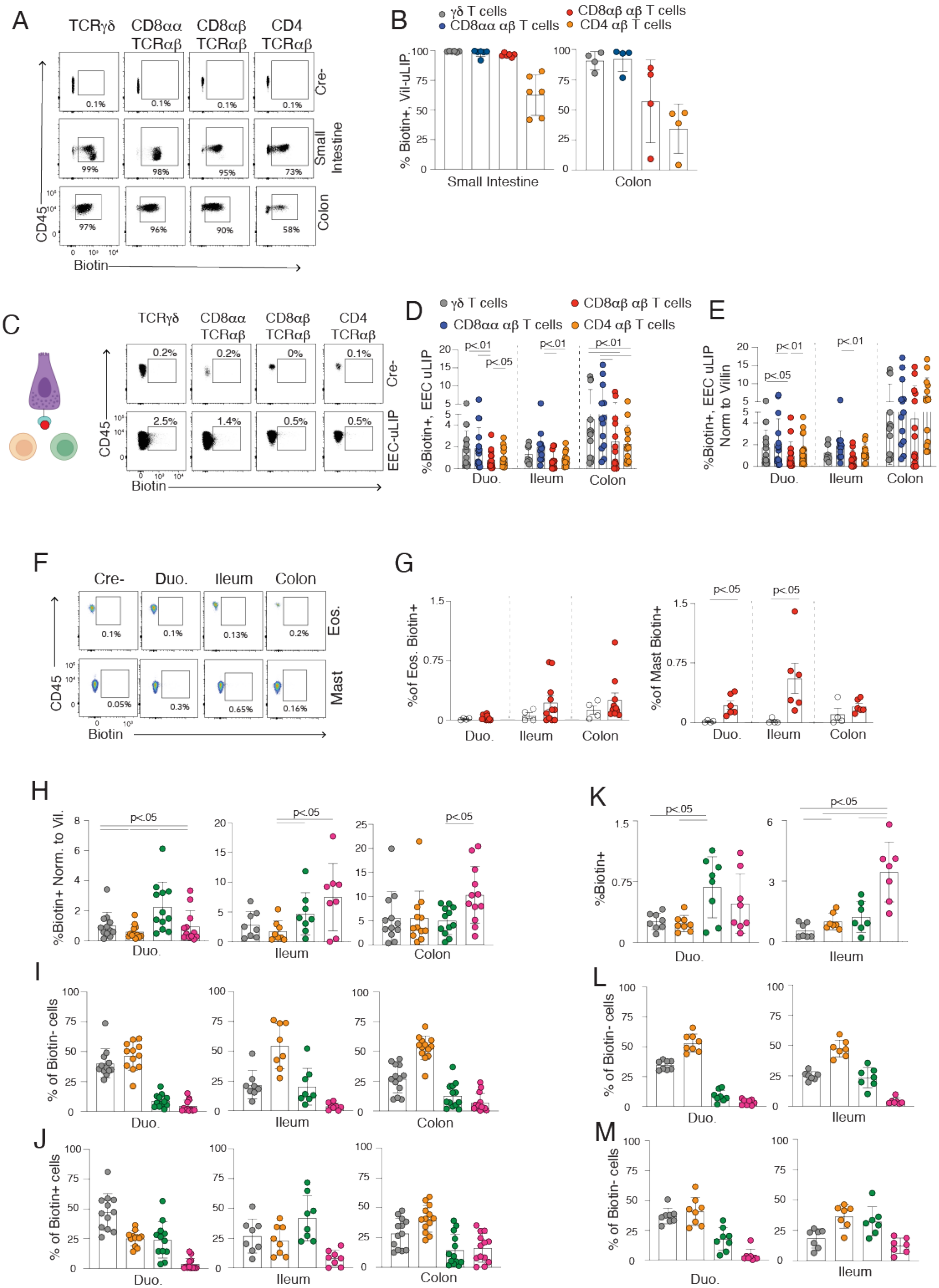

bioRxiv preprint doi: https://doi.org/10.64898/2026.06.04.730224; this version posted June 8, 2026. The copyright holder for this preprint (which was not certified by peer review) is the author/funder, who has granted bioRxiv a license to display the preprint in perpetuity. It is made available under a CC-BY 4.0 International license.

Supplemental Figure 4. Epithelial subset-specific interactions in steady state and after challenge. **A-B**. Villin-uLIPSTIC analysis at the baseline. **A**. Representative FACS plots showing Biotin+ γδ T cells (CD45+, TCRγδ+, TCRβ-), CD8αα T cells (CD45+, TCRγδ-, TCRβ+, CD8α+, CD8β-, CD4-), CD8αβ T cells (CD45+, TCRγδ-, TCRβ+, CD8α+, CD8β+, CD4-), and CD4 T cells (CD45+, TCRγδ-, TCRβ+, CD8β-, CD4+). **B**. Quantification from n=4 mice. **C-E**. Neurod1-uLIPSTIC (enteroendocrine cell-specific) analysis at baseline. **C**. Representative flow cytometry plots. **D**. Quantification of Biotin+ immune cells. **E**. Normalization to Villin-uLIPSTIC data. **F-G**. Analysis of Pou2f3-uLIPSTIC (tuft cell-specific) after sixth OVA-OC. F. Representative FACS plot showing Biotin+ mast cells (CD45+, TCRγδ-, TCRβ-, Fcer1+, cKIT+) and eosinophils (CD45+, TCRγδ-, TCRβ-, CD11b+, SiglecF+). **G**. Quantification. **H-M**. Neurod1-uLIPSTIC and Defa6-uLIPSTIC (Paneth cell-specific) analysis after sixth OVA-OC. **H, K**. Biotin+ normalized to Villin-uLIPSTIC data for the indicated iEC subset uLIPSTIC strains. **I, J, L, M**. Percentage of total biotin-negative (**I, L**) or biotin-positive (**J, M**) cells that are the indicated immune cell type; these percentages were used to calculate the interaction scores shown in Figure 5L and 5O. T-test with Welch's correction or one-way ANOVA with Tukey's multiple comparisons correction was performed where applicable for panels D, E, G, and H-M. Each dot in the bar graphs represents a biological replicate derived from a unique mouse, and the error bars represent the standard deviation.

bioRxiv preprint doi: https://doi.org/10.64898/2026.06.04.730224; this version posted June 8, 2026. The copyright holder for this preprint (which was not certified by peer review) is the author/funder, who has granted bioRxiv a license to display the preprint in perpetuity. It is made available under a CC-BY 4.0 International license.

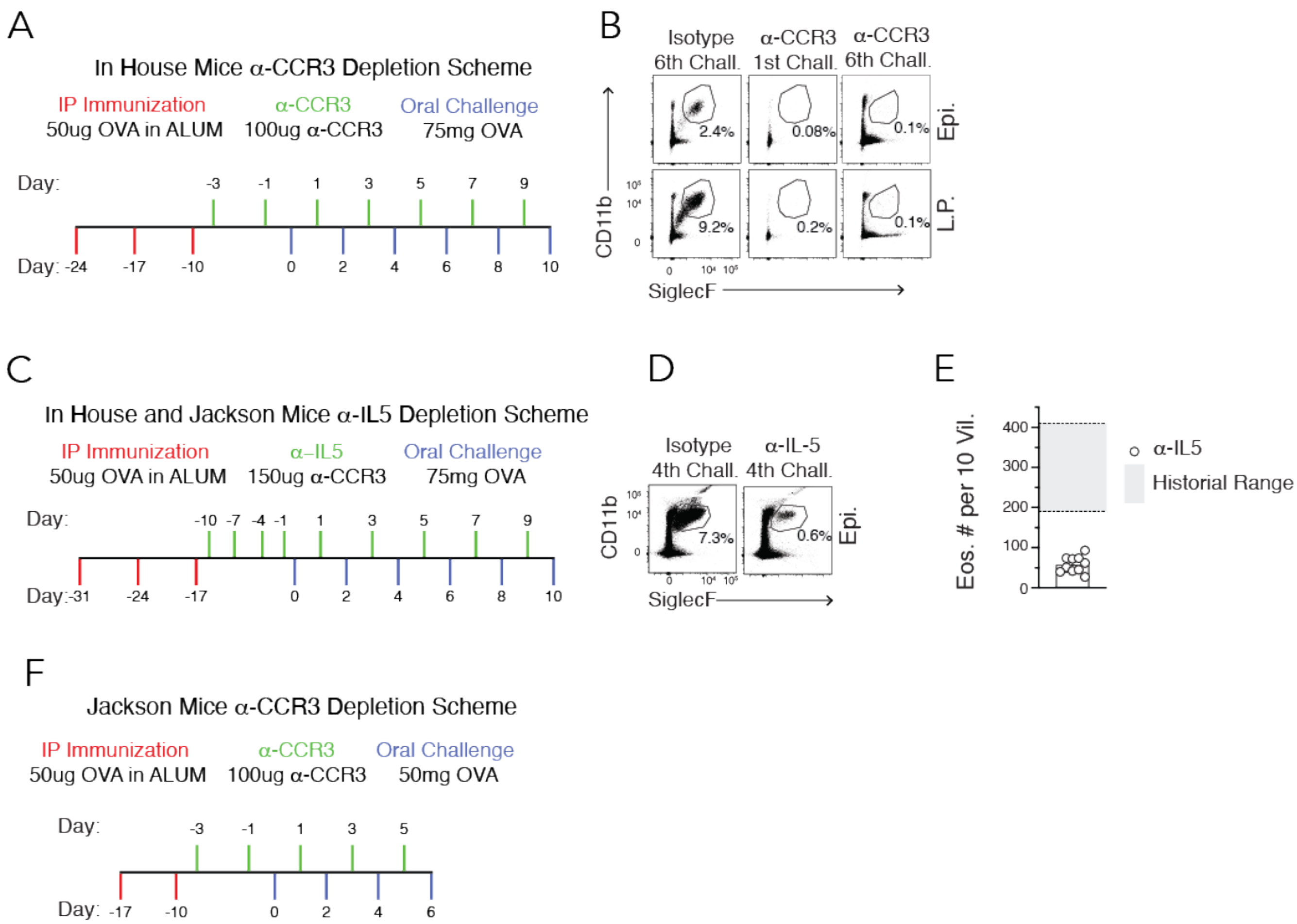

Supplemental Figure 5. Eosinophil depletion validation and kinetics. **A, C, F**. Scheme representing of antibody treatment, sensitization, and oral challenge protocols for anti-CCR3 (**A, C**) and anti-IL-5 (**F**) eosinophil depletion approaches. Mice were sensitized by intraperitoneal injection of OVA-Alum weekly for three weeks and then treated with depleting antibodies prior to and throughout the challenge phase. **B, D**. Flow cytometry analysis of eosinophil depletion efficiency at the indicated time points using anti-CCR3 (**B**) and anti-IL-5 (**D**) treatments. Representative FACS plots showing biotin labeling of eosinophils in treated versus isotype control mice. **E**. Quantification of eosinophil numbers per 10 villi in anti-IL-5 treated mice at the indicated challenge timepoints, demonstrating dose-dependent depletion efficiency.
